# Nicotine as a cardiovascular drug: haemodynamic stress, oxidative injury, and the regulatory failure to protect the heart

**DOI:** 10.1093/ehjopen/oeag065

**Published:** 2026-05-18

**Authors:** Thomas Münzel, Filippo Crea, Sanjay Rajagopalan, Thomas F Lüscher

**Affiliations:** Department of Cardiology, University Medical Center Mainz, Johannes Gutenberg University, Langenbeckstrasse 1, Mainz 55131, Germany; Department of Cardiovascular Medicine, Catholic University of the Sacred Heart, Rome 00168, Italy; Harrington Heart and Vascular Institute, University Hospitals Cleveland Medical Center, Cleveland, OH, USA; Center for Molecular Cardiology, University of Zurich, 8952 Schlieren, Switzerland; Heart Division, Royal Brompton and Harefield Hospitals, London SW3 6NP, UK; National Heart and Lung Institute, Imperial College London, London SW3 6LY, UK; School of Cardiovascular and Metabolic Medicine & Sciences, King’s College London, London SE1 1UL, UK

**This correspondence refers to ‘Nicotine and the cardiovascular system: unmasking a global public health threat’, by T Münzel *et al.*  https://doi.org/10.1093/eurheartj/ehaf1010.**

**Authors' response to the commentary of Miguel and Steffensen, https://doi.org/10.1093/ehjopen/oeag059.**

We thank Miguel and Steffensen for their correspondence^1^ and the opportunity to clarify what our paper does, and does not, claim. Our central message aligns with broad cardiovascular consensus: eliminating combustion can reduce exposure to several toxicants, but it does not confer cardiovascular ‘safety’ for nicotine-containing consumer products.^1,2^ This position is consistent with statements and scientific assessments from the American Heart Association (AHA), the European Society of Cardiology (ESC), the World Health Organization (WHO), the World Heart Federation (WHF), the European Environment Agency (EEA), and the U.S. Centers for Disease Control and Prevention (CDC), all of which recognize nicotine’s addictive potential and its adverse cardiovascular effects. Put simply: ‘less harmful’ is not ‘harmless.’

**1) Exposure matrices differ, yet nicotine remains a biologically active cardiovascular stressor across delivery systems**

We agree that product-specific emission profiles, routes of administration, and pharmacokinetics matter. We did not argue that all nicotine products are equivalent, and we explicitly identified combustible cigarettes as the most harmful category.^2^

However, product differentiation does not negate nicotine’s conserved cardiovascular actions. Nicotine is a potent autonomic drug: it activates nicotinic acetylcholine receptors, increases catecholamine release, raises heart rate and blood pressure, and promotes vasoconstriction and haemodynamic load, effects that run counter to the aims of cardiovascular prevention and therapy.^3^ These haemodynamic effects are clinically meaningful stress signals, particularly in patients with atherosclerosis or structural heart disease.

Importantly, nicotine is a highly addictive substance with dependence liability comparable to heroin and cocaine, mediated through rapid dopaminergic activation of mesolimbic reward pathways and powerful cue-conditioning dynamics.^4^ This addictive potential fundamentally alters the public-health calculus, especially in adolescents, whose brains remain in active neurodevelopment and are particularly vulnerable to long-term dependence trajectories.

**2) Acute haemodynamic triggers and mechanistic injury are clinically relevant, even as long-term outcome evidence continues to evolve**

Miguel and Steffensen argue that acute responses and biomarker changes cannot support clinical inference. That position is inconsistent with cardiovascular science, where acute sympathetic surges, blood-pressure spikes, and tachycardia are accepted precipitants of myocardial infarction, arrhythmias, and stroke in susceptible individuals.^5^

Mechanistically, nicotine is not merely a shared constituent; it can act as a direct vascular toxin. Experimental evidence shows nicotine impairs endothelial nitric oxide (NO) signalling via BH_4_-related pathways and eNOS uncoupling, accelerating endothelial dysfunction and atherosclerosis in vivo.^6^

This observation is highly important, since nicotine is able to switch the antiatherosclerotic NO producing, antiatherosclerotic enzyme to a proatherosclerotic superoxide producing enzyme.^7^

Endothelial dysfunction is an integrative downstream phenotype linking haemodynamic stress, oxidative injury, and event risk. Importantly, endothelial dysfunction predicts adverse cardiovascular outcomes in coronary artery disease cohorts.^8^

**3) Observational e-cigarette associations: we acknowledge confounding, this supports precaution, not reassurance**

We agree that many e-cigarette datasets are entangled with prior smoking, dual use, and temporality limitations. This is precisely why we argued that long-term adjudicated outcomes, especially in nicotine-naïve users, remain the key evidence gap.^2^

However, uncertainty is not a safety signal. Where exclusive-use cohorts are small and follow-up is limited, the absence of definitive myocardial infarction or stroke signals in current datasets should therefore not be interpreted as evidence of neutrality, but rather reflects the limited duration, sample size, and residual confounding present in many of these studies. Large population datasets demonstrate that dual use frequently yields inflammatory and oxidative biomarker profiles comparable to smoking, consistent with a continuum of harm in which incomplete switching nullifies potential benefit.^9^

**4) Comment on the cited RCT meta-analysis: short-term NRT trials cannot be generalized to chronic high-dose consumer nicotine**

The correspondence cites a systematic review of randomized trials reporting no association between nicotine and major cardiovascular events.^10^ This evidence requires contextualization.

Most included trials evaluate medically supervised, time-limited nicotine replacement therapy rather than chronic high-dose consumer exposure.

Cardiovascular events are rare and not primary endpoints; follow-up is limited; confidence intervals remain compatible with modest risk increases.

Furthermore, NRT delivers nicotine slowly and at lower peak concentrations, unlike many modern consumer products engineered for rapid systemic exposure.

These trials may be reassuring for therapeutic NRT under supervision, but they do not establish the cardiovascular safety of long-term, high-exposure nicotine consumption in consumer markets.

**5) Snus and oral nicotine: outcome signals exist, and hypertension is not a ‘surrogate’, it is a causal disease pathway**

For smokeless tobacco, outcome signals are not hypothetical. Snus use has been associated with incident hypertension in prospective settings, consistent with nicotine’s sympathomimetic effects.^11^

Crucially, in patients with established cardiovascular disease, continued snus use after myocardial infarction has been associated with increased all-cause and cardiovascular mortality, demonstrating a clinically consequential risk in post-MI patients and directly relevant for secondary prevention.^12,13^ Broader pooled cohort analyses confirm increased mortality among persistent snus users.^14^ For nicotine pouches, long-term outcome data remain limited; absence of evidence must not be communicated as evidence of safety, particularly given a rapid youth uptake and escalating nicotine-delivery efficiencies.^15^

**6) Transparency and certainty: our paper synthesizes convergent evidence to inform prevention under regulatory lag**

Miguel and Steffensen request evidence-graded methods and imply that without outcome trials across all products, strong prevention messages are inappropriate. The GRADE framework was developed to structure certainty of evidence and strength of recommendations, not to equate incomplete long-term outcome data with proof of safety.^16^ Under GRADE, observational evidence may begin at lower certainty but can be upgraded when biological plausibility and consistency converge; strong recommendations remain appropriate when harms are serious and exposure widespread.

Cardiovascular medicine has historically acted on convergent mechanistic and epidemiological evidence long before decades-long trials matured—for smoking and blood pressure.^17^ GRADE structures uncertainty; it does not transform uncertainty into reassurance.

Regulation has not kept pace with product innovation; youth addiction has become urgent. We therefore call for a comprehensive Nicotine Products Directive harmonizing standards across delivery systems.

**7) Nicotine is a drug: the regulatory paradox must be corrected**

Nicotine fulfils all criteria of a pharmacologically active drug: defined molecular targets, dose-dependent systemic effects, reproducible haemodynamic and oxidative cardiovascular injury, and substantial addiction liability.^3^

Yet nicotine is regulated as a consumer substance in high-dose delivery systems, while low-dose NRT is treated as medicinal. This regulatory paradox is untenable. In cardiology, raising heart rate and blood pressure via activation of the sympathetic nervous system is the opposite of what we prescribe to prevent events. A substance classified as a drug at low doses cannot rationally be treated as benign at higher doses and faster delivery.

**8) Industry affiliations and framing of evidence**

In the interpretation of studies on nicotine and cardiovascular risk, transparency regarding potential commercial interests is important, as such affiliations may influence how evidence is framed and communicated. While this does not invalidate scientific arguments, it warrants context when critiques echo long-standing industry narratives minimizing intrinsic toxicity and reframing uncertainty as safety.

Cardiovascular prevention cannot depend solely on randomized controlled trials (RCTs) for every single exposure before acting. Indeed, this is the case for smoking at large as RCTs were and are ethically impossible to implement. The association of nicotine with sympathetic activation and blood pressure elevation is strong and plausible as their causal role for outcomes is clear, and prevention is the ethical default. Thus, the same standards of biological plausibility and evidence convergence should apply to nicotine across all delivery systems.

## Conclusion

Combustion maximizes harm, but nicotine-driven haemodynamic stress, autonomic imbalance, oxidative vascular injury, and powerful addictive reinforcement remain biologically plausible and clinically relevant across delivery systems.^2^

In the light of converging mechanistic, epidemiological, and addiction data, immediate political action is warranted, meaning that until robust long-term outcomes, particularly in nicotine-naïve users, are available, the most cardiovascular-protective strategy remains preventing initiation (especially among youth) and supporting complete cessation. Regulatory inertia risks normalizing long-term addiction under the misconception of harm reduction. Cardiovascular prevention must remain grounded in biological evidence and the precautionary principle.
